# Mood-related behavioral and neurochemical alterations in mice exposed to low chlorpyrifos levels during the brain growth spurt

**DOI:** 10.1371/journal.pone.0239017

**Published:** 2020-10-02

**Authors:** Anderson Ribeiro-Carvalho, Carla S. Lima, Ana C. Dutra-Tavares, Fernanda Nunes, André L. Nunes-Freitas, Cláudio C. Filgueiras, Alex C. Manhães, Armando Meyer, Yael Abreu-Villaça

**Affiliations:** 1 Departamento de Ciências, Faculdade de Formação de Professores da Universidade do Estado do Rio de Janeiro, São Gonçalo, RJ, Brazil; 2 Instituto Federal de Educação, Ciência e Tecnologia do Rio de Janeiro, Rio de Janeiro, RJ, Brazil; 3 Laboratório de Neurofisiologia, Departamento de Ciências Fisiológicas, Instituto de Biologia Roberto Alcantara Gomes, Universidade do Estado do Rio de Janeiro, Rio de Janeiro, RJ, Brazil; 4 Instituto de Estudos em Saúde Coletiva e Faculdade de Medicina, Universidade Federal do Rio de Janeiro, Rio de Janeiro, RJ, Brazil; Weizmann Institute of Science, ISRAEL

## Abstract

Organophosphates are among the most used pesticides. Particularly, chlorpyrifos (CPF) is responsible for a number of deleterious effects on brain development, which may program behavioral changes later in life. Here, we investigated whether a regimen of early low level CPF exposure that did not result in a significant inhibition of acetylcholinesterase (AChE) had deleterious effects on mood-related behaviors, as well as on cholinergic and serotonergic biomarkers in the mice brain. From the 3^rd^ to 9^th^ postnatal day (PN), male and female Swiss mice were subcutaneously injected with CPF. Mice were submitted to a battery of behavioral tests from PN60 to PN63: open field, elevated plus maze and forced swimming tests. The cholinergic and serotonergic biomarkers were assessed at PN10 and PN63. Our data indicated that early CPF exposure increased anxiety-like behavior in females and altered decision-making behavior in both sexes. Most biochemical alterations were sex-dependent and restricted to females. At PN10, CPF female mice showed increased serotonin and choline transporter binding in cerebral cortex. Distinctively, in adult females, the effects indicated a hypoactive state: CPF exposure reduced 5-HT_1a_ receptor binding in cerebral cortex, as well as serotonin transporter binding and choline acetyltransferase activity in brainstem. Our results indicate that CPF exposure during the brain growth spurt deregulates serotonergic and cholinergic biomarkers. The effects are consistent with impaired synaptic function, may be related to long-term mood disorders and point out to higher female susceptibility.

## Introduction

The use of pesticide in Brazil increased drastically in the past few years [1–3]. In 2018, 450 new agrochemical licenses were issued by ANVISA (Brazilian Agency of Health Surveillance). In 2019, hundreds of additional pesticides were approved by the Brazilian government to use in agriculture [3, 4]. The utilization of some long-used agrochemicals also increased. Particularly, the use chlorpyrifos (CPF), an organophosphate (OP) insecticide that seems to be responsible for a number of deleterious effects on the nervous system, has more than doubled over the last decade in Brazil. In the USA, the Trump administration has recently rejected an Obama-era petition to ban this pesticide, against public opinion and scientific advice [5].

OPs cause changes in brain function that may culminate in psychiatric disorders [6, 7]. Epidemiological studies indicate that exposure to OPs is associated with increased rates of depression and suicide [8, 9]. Several neurotransmitter systems have been identified as responsible for the development of mood disorders, particularly of depression. Among them, the cholinergic system has been associated with the modulation of depression and anxiety (for review: Graef et al., [10]). In addition, the serotonergic system seems to play an important role and recent studies in animal models have identified, after exposure to OPs, changes in this system and behavioral deficits associated with mood disorders [11–13].

Acute exposure to high levels of chlorpyrifos involves irreversible inhibition of the enzyme acetylcholinesterase (AChE), resulting in the accumulation of acetylcholine in synaptic clefts and, consequently, cholinergic hyperstimulation [14]. However, the impact of exposure to low doses of pesticides is possibly even more relevant since subchronic or chronic exposures to low doses are more common in real life than high-level exposures.

There is evidence that the developing brain is particularly vulnerable to OP exposure, which is consistent with evidence that neurodevelopmental effects may occur at doses below the threshold for systemic toxicity or even for AChE inhibition [14, 15]. In a previous study, we described that early exposure to the OP methamidophos elicited late-emergent behavioral alterations, suggestive of increased depressive-like behavior, as well as cholinergic and serotonergic brain alterations [12]. These broad-spectrum effects corroborate previous evidence that OPs mechanisms of action are not restricted to AChE inhibition [14]. However, like in most other studies that address OP actions, the doses that were used, despite being lower than the threshold for systemic toxicity, evoked significant AChE inhibition, a finding that does not rule out the inhibition of this enzyme as a possible mechanism.

Rodent models have indicated that the brain growth spurt period is particularly susceptible to insults that lead to behavioral alterations later in life. This period occurs during early postnatal life in rodents and third trimester of gestation in humans. This period is characterized by a rapid increase in brain size, intense neurogenesis, cell migration, and synaptogenesis [16]. Here, we hypothesize that the brain growth spurt is a critical period concerning CPF-induced mood-related alterations on brain function, and that neurochemical and behavioral alterations would be identified even after a CPF dose that evokes negligible levels of AChE inhibition. To verify this hypothesis, we exposed neonatal mice to a subchronic low-level regimen of CPF exposure. We evaluated the effects CPF on mood using a battery of behavioral tests: open field, elevated plus maze and forced swimming tests. Considering the relevant association among the cholinergic system, the serotonergic system and mood disorders, we evaluated these two neurotransmitter systems in the cerebral cortex and brainstem both by the end of exposure and at adulthood. Regarding the cholinergic system, we assessed the binding of [3H] hemicholinium-3 (HC-3) to the high-affinity presynaptic choline transporter (Ch transporter) and the choline acetyltransferase (ChAT) activity. As for serotonergic system, we choose to assess 5-HT_1A_ and 5-HT_2_ receptors, and the presynaptic 5-HT transporter. In addition, since OPs exposure generates sex-selective consequences [14, 15], we included both male and female mice in the study.

## Materials and methods

All experiments were carried out under institutional approval of the Animal Care and Use Committee of the Universidade do Estado do Rio de Janeiro (Protocol number 0062011), in accordance with the Guide for the Care and Use of Laboratory Animals as adopted and promulgated by the National Institutes of Health and Brazilian Law nº 11.794. All mice were bred and maintained in a temperature-controlled environment, on a 12:12h light/dark cycle (lights on at 1:00 a.m.). Access to food and water was *ad lib*. All animal manipulations and behavioral tests were carried out in a sound-attenuated room adjoining the animal facility. Original breeding stock was obtained from Instituto Vital Brazil (Rio de Janeiro, RJ, Brazil).

After mating, each female mouse was placed in an individual cage. The day of parturition was considered postnatal day 1 (PN1). Thirty-five litters were submitted to the following experimental protocol. All pups from each litter were s.c. injected with chlorpyrifos (3 mg/Kg; CPF group) or to equivalent injections of the vehicle (CT group) every day, once daily, from PN3 to PN9. Chlorpyrifos was dissolved in dimethyl sulfoxide (vehicle) to provide adequate dilution and proper delivery. In order to avoid oversampling and minimize litter effects, data from males and females of the same litter were averaged separately within each experimental group. Body masses were measured daily during the period of exposure. Twenty-four animals were sacrificed four hours after the last injection at PN9 and the brains were dissected and the cerebral cortex and brainstem were immediately frozen and stored at -80°C for AChE activity analysis. At PN10, another thirty-three mice were used to evaluate the cholinergic and serotonergic systems. Seventy-four mice were maintained in the vivarium until adulthood, at which time they were submitted to a battery of behavioral tests from PN60 to PN63, after which animals were euthanized by decapitation. The brains were dissected and stored for later cholinergic and serotonergic systems analyses.

### Behavioral tests

From PN60 to PN61, mice were submitted to the three behavioral tests described below. Due to the presence of technical problems with the video data in some of the tests (recordings accidentally interrupted during the test and corrupted data during the process of transferring them to a computer), the sample size used for the quantitative analysis (indicated in the legends) varied from test to test. On the first day, anxiety-like behavioral levels were assessed in the elevated plus maze. This test was performed between 2:00 and 4:00 p.m. On the following day, mice were submitted to the open field test in the morning (between 9:00 and 11:00 a.m.) and to the forced swimming test in the afternoon (between 2:00 and 4:00 p.m.). The open field was used to assess locomotor activity, whereas the forced swimming test investigated the depressive-like behavior. Since the elevated plus maze and forced swimming tests are classically used to investigate emotional reactivity, both tests were performed during the same period of the circadian cycle of the mice (dark phase of the vivarium). The open field was performed in the light phase. All animals were allowed to habituate for 10 min in the testing room before each behavioral test and all behavioral tests were performed in a sound-attenuated testing room next to our vivarium and with lights on (60 W fluorescent light bulb, 3 m high) so that the mice could access the visual cues necessary to perform the tasks.

### Elevated plus maze (EPM)

Anxiety-like behavior was initially investigated by using the EPM test. The test procedure is described in detail elsewhere [17–19]. The EPM is shaped like a plus sign and consists of two “open” (no walls, 5 × 28.5 cm) and two “closed” (5 × 28.5 × 14 cm) arms, arranged perpendicularly and elevated 50 cm above the floor. The test began with the animal being placed on the center of the equipment, facing a closed arm. Each test lasted 10 min and all tests were videotaped. The percentage of open arms entries (%Entries OA: the number of entries in open arms divided by number of entries in open + closed arms) was used as an anxiety-like measure [20]. Increased %Entries OA corresponds to decreased anxiety-like state and vice versa [20]. The number of closed arms entries (Entries CA) was used as a measure of activity. In addition, the time spent in the center of the maze (Time Cen) was also measured. All variables were scored using the video images of the tests.

### Open field (OF)

The OF arena consists of a transparent acrylic box (46 cm length × 46 cm width × 43 cm height) equipped with 2 arrays of 16 infrared beams each, positioned at 1.5 cm above the floor, to measure horizontal spontaneous locomotor activity. Interruptions of photocell beams were detected by a computer system and the software, with a 0.1-s resolution, calculated the location of the animal. Each mouse was individually placed in the center of the arena, and spontaneous locomotor activity was determined. Locomotor activity was quantified on the basis of the traveled distance during a 10-min period.

### Forced swimming test (FST)

Each mouse was submitted to a 10-min FST session. The test procedure is described in detail elsewhere [21, 22]. Briefly, each mouse was placed in a plastic container (21 cm diameter × 23 cm height) filled with 16 cm of water at about 25°C. The animal’s behavior was continuously recorded throughout the testing session with an overhead video-camera. Animals were considered to be immobile when they remained floating with all limbs and tail motionless. The time the animals spent in this condition was used as a measure of depressive-like behavior. It should be noted, however, that the FST has limited capability of mimicking the depression traits present in humans, therefore, generalizations to the human population should be made with care.

### Evaluation of cholinergic and serotonergic systems

Mice were sacrificed at PN9 (4 h after the last injection), PN10 or PN63. Brain dissection was performed by a cut through the cerebellar peduncles, whereupon the cerebellum (including flocculi) was lifted from the underlying tissue. The cerebral cortex (forebrain with removal of the hippocampus) was separated from the brainstem (midbrain + pons + medulla) by a cut made rostral to the thalamus. All tissue collection was done between 2:00 and 4:00 p.m., immediately after the last behavioral test in adult animals. In all experiments, proteins were measured by bicinchoninic acid (BCA) protein assay.

Regarding the cholinergic system, we evaluated the AChE activity, the choline acetyltransferase (ChAT) activity and the binding of [^3^H]hemicholinium-3 (HC-3) to the high-affinity presynaptic choline transporter (Ch transporter). AChE is the enzyme that catalyzes the hydrolysis of acetylcholine to choline and acetate. ChAT, the enzyme that catalyzes acetylcholine biosynthesis, is a constitutive marker of cholinergic synapses, which reflects the concentration of cholinergic nerve terminals [23–25]. In contrast, the Ch transporter, which transports choline to the presynaptic terminal, is responsive to neuronal activity [26].

AChE activity was measured using a spectrophotometric method described by Ellman [27]. The cerebral cortex and the brainstem of each animal were weighed and homogenized to approximately 90 mg/mL in sodium phosphate buffer (0.12 M, pH 7.6) using a homogenizer Ultra-Turrax T10 basic (IKA, São Paulo, SP). Each assay contained 0.1 mL of diluted homogenate in a total volume of 1.16 mL with final concentrations of 102 mM of sodium phosphate buffer (pH 7.6), 0.3 mM of 5,5-dithiobis(2-nitrobenzoic) acid (DTNB) and 1 mM of acetylthiocholine iodide. Immediately after the addition of tissue, the duplicates were read at 412 nm in kinetic mode every 30 s during 2 min. Blank’s absorbances were subtracted from the final readings. To get the values of AChE activity in nmols/min, we used a previously built standard curve of L-cysteine. The activity was determined relative to tissue protein.

For all other analysis, tissues were thawed and homogenized in ice-cold 50 mM Tris (pH 7.4) using a homogenizer Ultra-Turrax type T10 basic. Aliquots of this homogenate were withdrawn for measurements of ChAT activity and total protein. The remaining homogenate was then sedimented by centrifugation at 39,000×g for 15 min. The pellet was resuspended (Ultra-Turrax) in the original volume of buffer, resedimented, and the resultant pellet was resuspended in ¼ of the original volume using a smooth glass homogenizer fitted with a Teflon pestle. Aliquots of this last resuspension were obtained for measurements of and [^3^H]HC-3 binding to the Ch transporter, 5-HT_1A_ and 5-HT_2_ receptor binding, 5-HT transporter binding and for membrane protein assessment. Proteins were measured by bicinchoninic acid (BCA) protein assay. All assays have been described in detail in previous papers [11, 12, 24, 25, 28, 29] and will therefore be presented briefly.

#### Choline acetyltransferase activity

To assess ChAT activity, assays containing tissue homogenate diluted in phosphate buffer (pH = 7.9), a mixture with final concentrations of NaCl 200 mM, MgCl_2_ 17 mM, EDTA 1 mM, Triton X-100 0.2% in buffer, physostigmine 0.12 mM, bovine serum albumin 0.6 mg/mL, choline chloride 20 mM and [14C]acetyl-coenzyme A 50 mM. Triplicate samples from each homogenate were pre-incubated for 15 min at 4ºC and then incubated for 30 min at 37ºC. Under these conditions, the enzymatic reaction took place and ChAT catalyzed the synthesis of acetylcholine. Labeled acetylcholine was then extracted and the activity determined relative to tissue protein.

#### High affinity choline uptake

The binding of HC-3 was determined using a final ligand concentration of 2 nM in the membrane fraction; incubations lasted 20 min at 20ºC in a buffer consisting of 10 nM NaKHPO4 / 150 nM NaCl (pH 7.4); unlabeled HC-3 20 μM was used to displace specific binding for the cholinergic transporter. Incubations were stopped by the addition of excess of ice-cold incubation buffer and the labeled membranes were trapped by rapid vacuum filtration onto glass fiber filters that were presoaked in 0.15% polyethyleneimine. The filters were then washed with incubation buffer and radiolabel was determined. Data were obtained by calculating the specific binding per mg of membrane protein.

For the serotonergic system analysis, we choose the 5-HT_1A_ and 5-HT_2_ receptors, two receptors that play major roles in 5-HT-related mental disorders, especially depression [30, 31], and the presynaptic 5-HT transporter, the primary target for antidepressant drugs [32].

#### Serotonin receptors and transporter

The 5-HT receptors binding were evaluated by using two radioligands: 1 nM [3H]8-hydroxy-2-(di-n-propylamino)tetralin for the 5-HT_1A_ receptors, and 0.4 nM [3H]ketanserin for the 5-HT_2_ receptors. Binding to the presynaptic 5-HT transporter was evaluated with 85 pM [3H]paroxetine. For the 5-HT_1A_ receptors, incubations lasted for 30 min at 25ºC in a buffer consisting of 50 mM Tris (pH 8), 0.5 mM MgCl_2_, and 0.5 mM sodium ascorbate; 100 μM 5-HT was used to displace specific binding. For the 5-HT_2_ receptors, incubations lasted 15 min at 37ºC in 50 nM Tris (pH 7.4) and specific binding was displaced with 10 μM methysergide. For the binding to the presynaptic 5-HT transporter, incubations lasted for 120 min at 20ºC in a buffer consisting of 50 mM Tris (pH 7.4), 120 mM NaCl, and 5 mM KCl; 100 μM 5-HT was used to displace specific binding. Incubations were stopped by the addition of ice-cold incubation buffer and the labeled membranes were trapped by rapid vacuum filtration onto glass fiber filters that were presoaked in 0.15% polyethyleneimine. The filters were then washed with incubation buffer and radiolabeling was determined. Data were obtained by calculating the specific binding per mg of membrane protein.

## Materials

Radioisotopically-labeled compounds came from PerkinElmer Life Sciences (Boston, MA): [^14^C] Acetyl-CoA (specific activity 4.0 Ci/mmol), [^3^H] HC-3 (specific activity 170 Ci/mmol), [^3^H]8-hydroxy-2-(di-n-propylamino)tetralin (specific activity 170.2 Ci/mmol), [^3^H]ketanserin (specific activity 67.0 Ci/mmol) and [^3^H]paroxetine (specific activity, 24.4 Ci/mmol). Sigma Chemical Co. (St. Louis, MO) was the source for bovine albumin, BCA kit, eserine hemisulfate salt, 3-heptanone, sodium tetraphenylborate, Triton X-100, chlorpyrifos (analytical standard, CAS 45395), serotonin, acetylthiocholine, methysergide and polyethyleneimine. VETEC Química Fina Ltda (Rio de Janeiro, RJ) was the source for all other reagents.

### Statistical analysis

Repeated measures analyses of variance (rANOVA) were performed for body mass during the treatment period. In order to minimize the influence of litter effects, we considered the average of values from mice of the same litter instead of using individual values. Treatment (CT or CPF) was used as between-subjects factor. Day was considered the within-subjects factor. For behavioral variables, univariate analyses of variance (uANOVAs) were carried out. Treatment (CT or CPF) and Sex were used as between-subjects factors. Whenever applicable, significant Treatment interactions were followed by lower-order ANOVAs.

For cholinergic and serotonergic markers, results were evaluated first by two rANOVAs on all factors: Treatment (CT or CPF), Brain Region (cerebral cortex and brainstem), Age (PN10 and PN63), and Sex. For the first rANOVA, cholinergic measures (ChAT and Ch transporter) were considered the within-subject factor. For the second rANOVA, serotonergic measures (5HT_1A_ receptor, 5HT_2_ receptor, and 5HT transporter) were considered the within-subject factor. Whenever the ANOVAs indicated effects that differed among the different within-subject factors, treatments, brain regions, ages, and/or sexes, data were then re-examined separately using lower order ANOVAs.

All data were compiled as means and standard errors. Significance was assumed at the level of *p* < 0.05 for main effects; however, for interactions at *p* < 0.1, we also examined whether lower-order main effects were detectable after subdivision of the interactive variables. The criterion for interaction terms was not used to assign significance to the effects but rather to identify interactive factors requiring subdivision for lower-order tests of main effects of Treatment, the factor of chief interest [33].

## Results

### Body mass and AChE activity

Body mass (Table 1) increased during the period of exposure in both experimental groups (Day: *F*_(1.3,70.7)_ = 816.5, *p* < 0.001). There were no significant Treatment effects.

**Table 1:** Body mass (grams).

	CT	CPF
**PN3**	2.40 ± 0.05	2.37 ± 0.06
**PN4**	2.81 ± 0.05	2.85 ± 0.08
**PN5**	3.29 ± 0.07	3.26 ± 0.08
**PN6**	3.71 ± 0.07	3.70 ± 0.10
**PN7**	4.13 ± 0.09	4.14 ± 0.12
**PN8**	4.57 ± 0.11	4.53 ± 0.12
**PN9**	4.90 ± 0.12	4.93 ± 0.16

CT = control group; CPF = chlorpyrifos-exposed group; PN = postnatal day.

As shown in Table 2, the selected regimen of developmental CPF exposure failed to evoke significant alterations in AChE activity in the cerebral cortex and in the brainstem both by the end of the period of exposure and at adulthood. Considering that AChE inhibition is considered as a toxicity biosensor for OP, our findings confirm that the CPF dose used in the current study was indeed low. There were no differences in AChE activity between males and females.

**Table 2:** AChE activity (nmoles/min/mg).

		CT	CPF
PN9	Cerebral Cortex	103.1 ± 17.6	90.2 ± 13.4
	Brainstem	361.8 ± 34.6	311.7 ± 24.2
PN63	Cerebral Cortex	747.4 ± 25.4	742.3 ± 25.8
	Brainstem	474.3 ± 14.7	500.9 ± 19.3

CT = control group; CPF = chlorpyrifos-exposed group; PN = postnatal day.

### Behavioral effects of CPF

**Fig 1:**
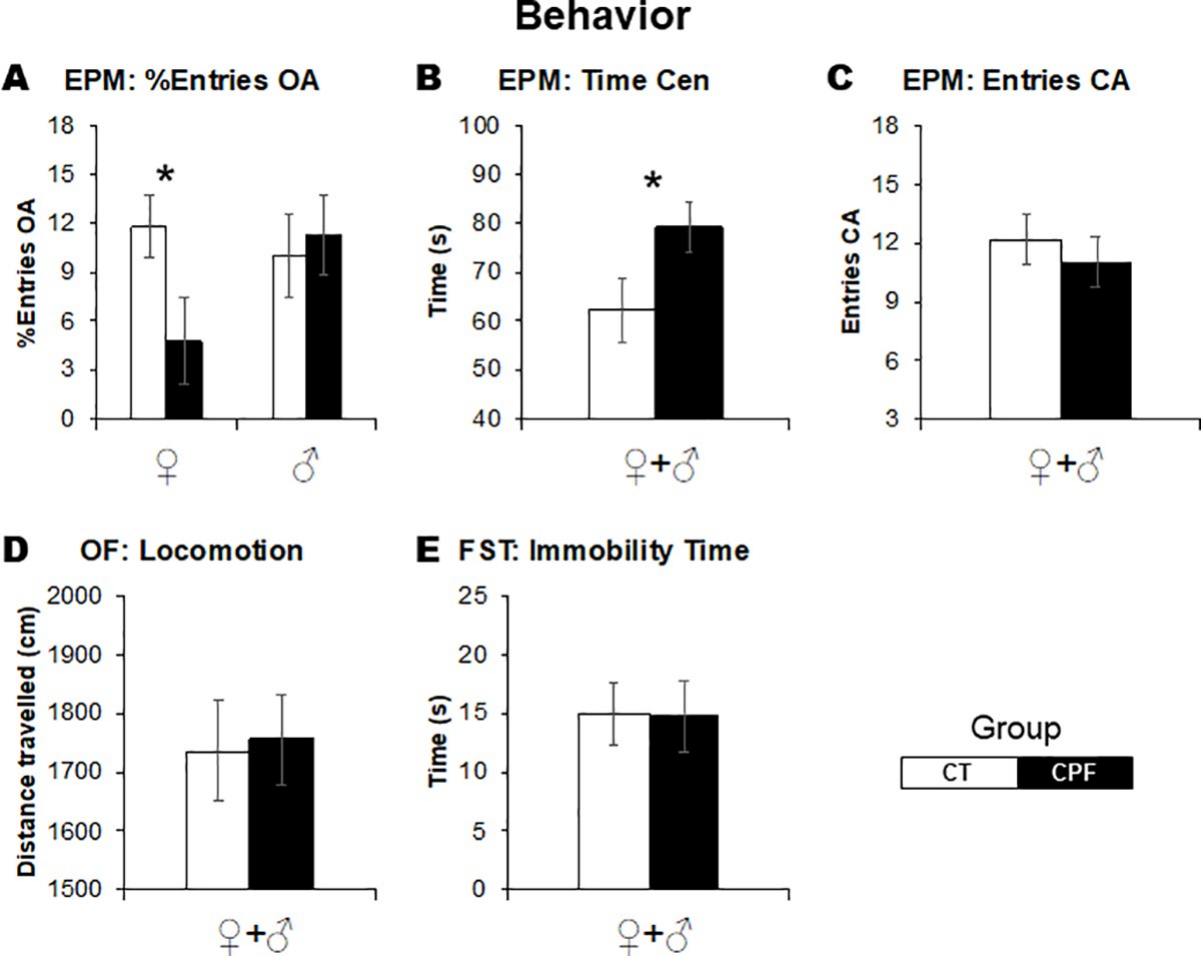
Effects of CPF exposure during the brain growth spurt (3 mg/Kg/day s.c., from PN3 to PN9) on behavior at adulthood. In (A), anxiety-like measure (%Entries OA) in the EPM. In (B), decision making (Time Cen), and, in (C), locomotor activity (Entries CA) measures in the EPM. In (D), locomotor activity (Ambulation) in the OF. In (E), immobility time in the FST. EPM, elevated plus maze. OF, open field. FST, forced swimming test. For each treatment group, 14–19 animals were examined, divided into males and females. White bars represent the control group and gray bars represent the CPF-exposed ones. Values are means ± SEM. *p < 0.05 versus respective control group.

In the EPM, the effect of neonatal CPF exposure on anxiety-like levels was sex-dependent (*Treatment x Sex*: *F*_(1,54)_ = 3.0, *p* = 0.08). Separate analysis for females and males indicated that, in females, CPF reduced the entries in the open arms of the EPM (%Entries OA; *Treatment*: *F*_(1,30)_ = 4.7, *p* = 0.03), which indicates increased anxiety-like behavior (Fig 1A). There were no significant effects in males (Fig 1A). Exposure to CPF increased the time spent in the center of the maze in both sexes (Time Cen; *Treatment*: *F*_(1,54)_ = 4.5, *p* = 0.03, Fig 1B), suggesting increased time spent in decision-making behavior. There were no significant effects on locomotor activity in EPM and OF (Fig 1C and 1D, respectively) as well as no alterations on behavior in the FST (Fig 1E).

### Overall analysis of cholinergic and serotonergic markers

The rANOVA across cholinergic biomarkers (ChAT and Ch transporter), treatments, brain regions, ages, and sexes identified interactions of Treatment × Age × Sex (*F*_(1,145)_ = 9.0, *p* = 0.03), Treatment × Region × Sex (*F*_(1,145)_ = 2.8, *p* = 0.098), Treatment × Cholinergic measures × Sex (*F*_(1,145)_ = 3.3, *p* = 0.085), Cholinergic measures × Region (*F*_(1,145)_ = 259.9, *p* < 0.001), and Cholinergic measure × Age (*F*_(1,145)_ = 367.0, *p* < 0.001). The rANOVA across the serotonergic biomarkers (5HT_1A_ receptor, 5HT_2_ receptor, and 5HT transporter), treatments, brain regions, ages and sexes identified interactions of Treatment × Serotonergic measures × Region × Sex (*F*_(2,238)_ = 4.3, *p* = 0.014), Treatment × Serotonergic measure × Age × Sex (*F*_(2,238)_ = 3.9, *p* < 0.021), Treatment × Serotonergic measures (*F*_(2,238)_ = 2.5, *p* = 0.083), Treatment × Serotonergic measure × Age (*F*_(2,238)_ = 3.0, *p* = 0.052), Serotonergic measure × Region (*F*_(2,238)_ = 260.4, *p* < 0.001), and Serotonergic measure × Age (*F*_(2,238)_ = 127.3, *p* < 0.001). Given the interactions identified in both rANOVAs, we separated the data into the individual cholinergic and serotonergic measures, brain regions and ages and then re-examined the results. After subdividing the data, we kept the factor Sex in the analysis.

### Effects on the cholinergic system

**Fig 2:**
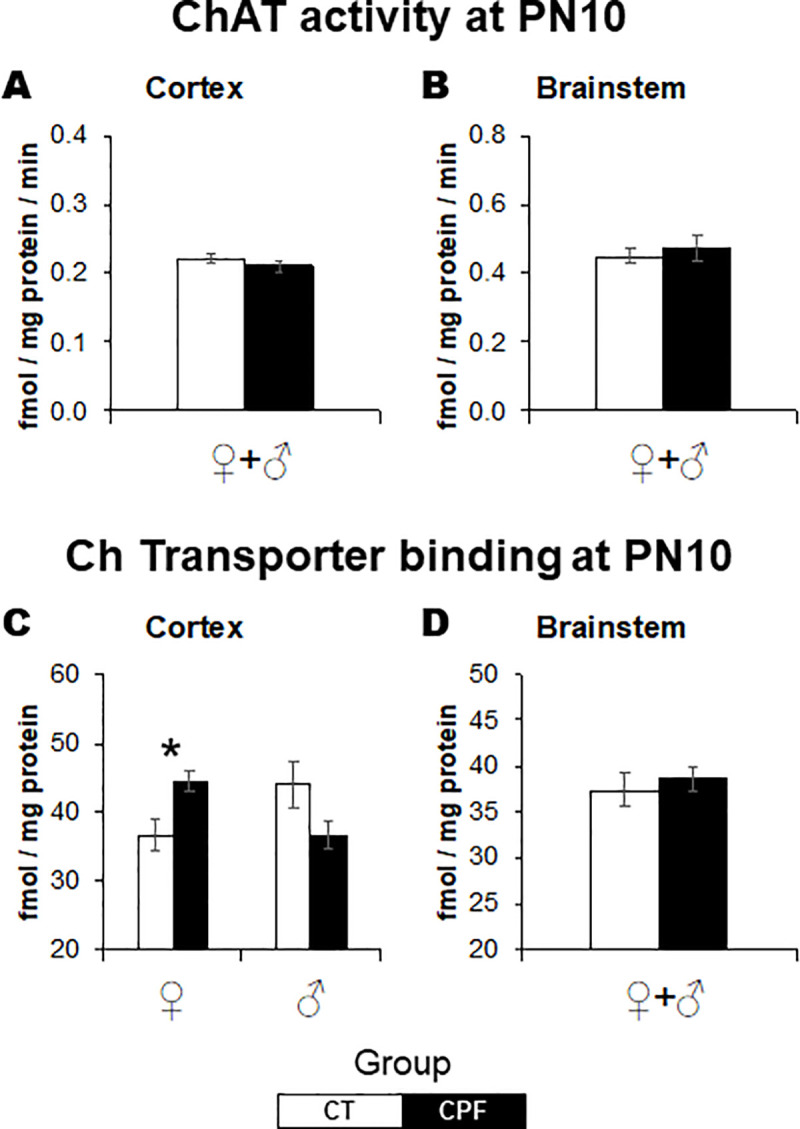
Effects of CPF exposure during the brain growth spurt (3 mg/Kg/day s.c., from PN3 to PN9) on the cholinergic system at PN10. ChAT activity in the cerebral cortex (A) and brainstem (B), and Ch transporter binding in the cerebral cortex (C) and brainstem (D). ChAT, choline acetyltransferase. Ch, choline. For each treatment group, 15–17 animals were examined, divided into males and females. White bars represent the control group and gray bars represent the CPF-exposed ones. Values are means ± SEM. *p < 0.05 versus respective control group.

While at PN10, CPF failed to evoke significant alterations in ChAT activity (Fig 2A and 2B), there were sex-selective effects of CPF in cortical Ch transporter binding (*Treatment x Sex*: *F*_(1,29)_ = 9.9, *p* = 0.004). In females (*Treatment*: *F*_(1,15)_ = 8.3, *p* = 0.012), CPF increased HC-3 binding to the Ch transporter (Fig 2C). There were no significant effects in males (Fig 2C).

**Fig 3:**
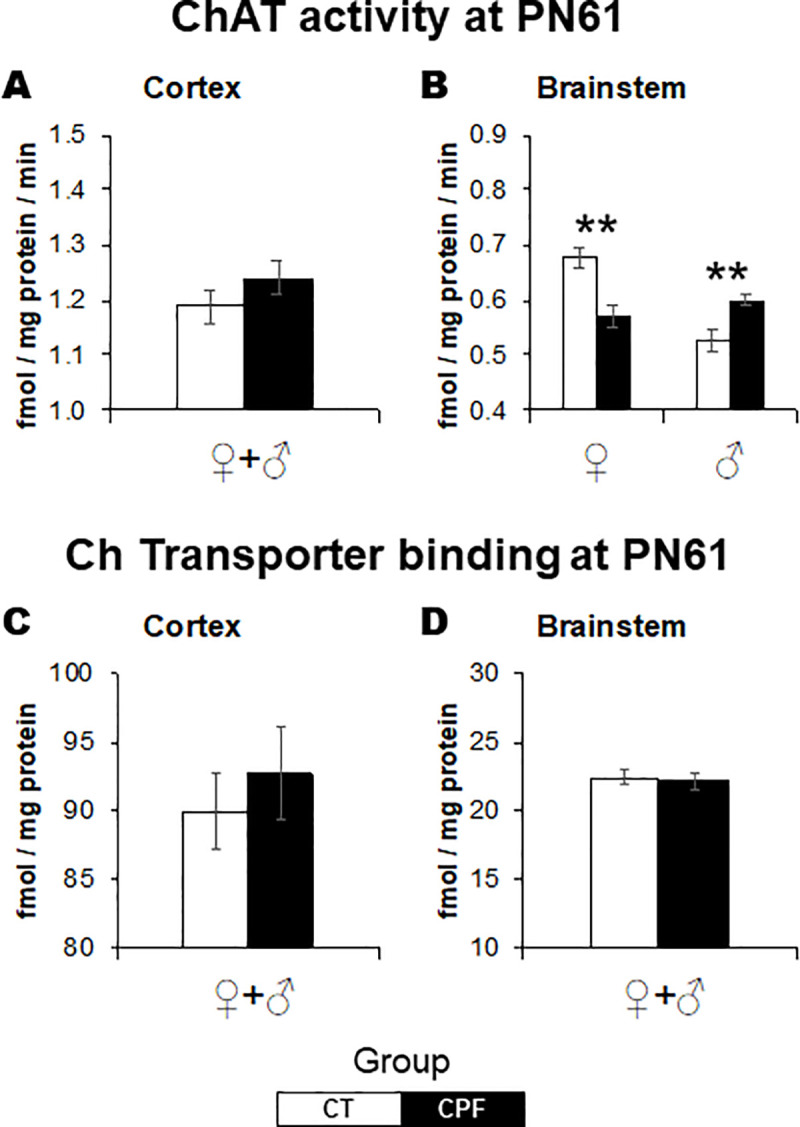
Effects of CPF exposure during the brain growth spurt (3 mg/Kg/day s.c., from PN3 to PN9) on the cholinergic system at adulthood. ChAT activity in the cerebral cortex (A) and brainstem (B), and Ch transporter binding in the cerebral cortex (C) and brainstem (D). ChAT, choline acetyltransferase. Ch, choline. For each treatment group, 16–17 animals were examined, divided into males and females. White bars represent the control group and gray bars represent the CPF-exposed ones. Values are means ± SEM. *p < 0.05 versus respective control group.

Neonatal CPF exposure elicited a late-emergent sex-dependent effect on ChAT activity in the brainstem (*Treatment x Sex*: *F*_(1,29)_ = 25.5, *p* < 0.001). Separate analyses for adult males and females indicated opposite effects. While CPF reduced ChAT activity in adult females (*Treatment*: *F*_(1,15)_ = 16.2, *p* = 0.001), in males (*Treatment*: *F*_(1,15)_ = 9.4, *p* = 0.008), ChAT activity was increased (Fig 3B). There were no differences in the cerebral cortex (Fig 3A). As for the Ch transporter, there were no lasting effects of early CPF exposure in both studied regions (Fig 3C and 3D).

### Effects on the serotonergic system

**Fig 4:**
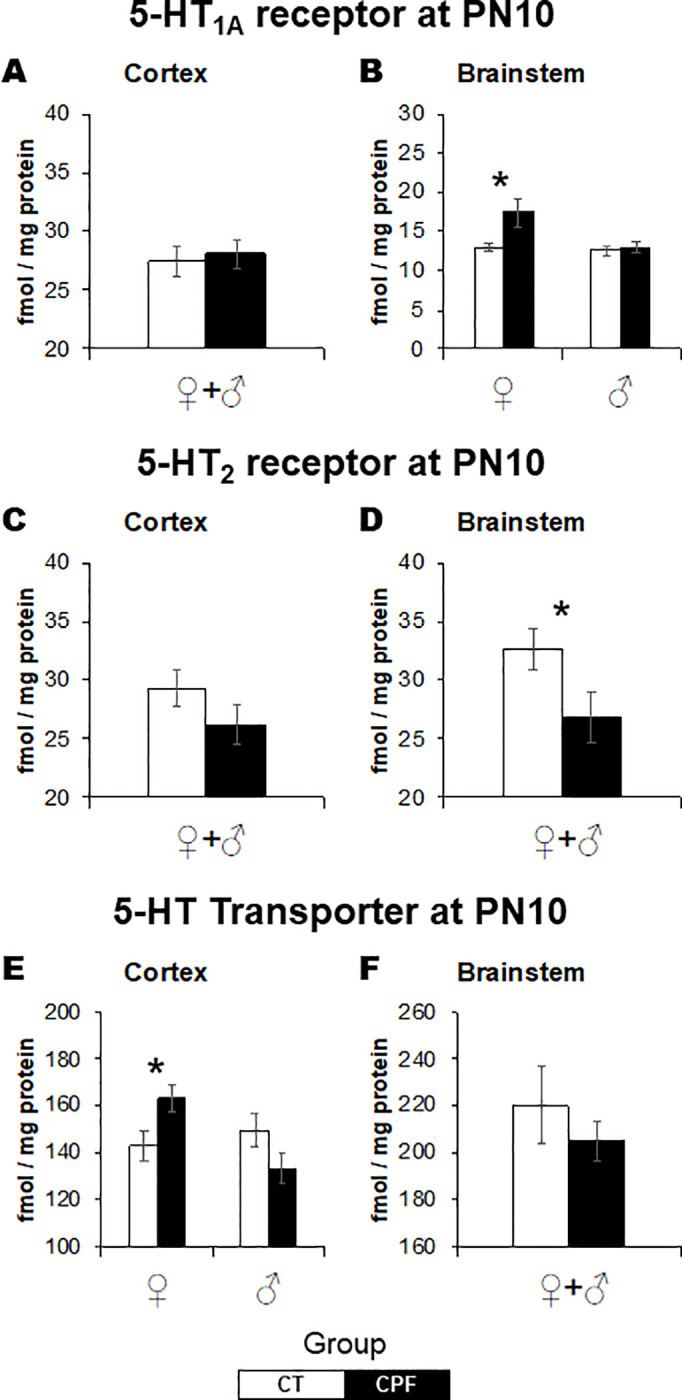
Effects of CPF exposure during the brain growth spurt (3 mg/Kg/day s.c., from PN3 to PN9) on the serotonergic system at PN10. 5-HT1a in the cerebral cortex (A) and brainstem (B), 5-HT2 in the cerebral cortex (C) and brainstem (D) and 5-HT transporter in the cerebral cortex (E) and brainstem (F). 5-HT1a, serotonin receptor subtype 1a. 5-HT2, serotonin receptor subtype 2; 5-HT, serotonin. For each treatment group, 11–12 animals were examined, divided into males and females. White bars represent the control group and gray bars represent the CPF-exposed ones. Values are means ± SEM. *p < 0.05, versus respective control group.

Neonatal CPF exposure also elicited significant alterations in the serotonergic system (Fig 4). At PN10, CPF exposure altered 5-HT_1a_ receptor (*Treatment x Sex*: *F*_(1,19)_ = 4.3, *p* = 0.050) and 5-HT transporter (*Treatment x Sex*: *F*_(1,18)_ = 7.2, *p* = 0.015) bindings in a sex-selective way. There were female-only increases in 5-HT_1a_ receptor binding in the brainstem (Fig 1B, *Treatment*: *F*_(1,10)_ = 6.3, *p* = 0.029) and in 5-HT transporter binding in the cerebral cortex (Fig 1E, *Treatment*: *F*_(1,8)_ = 5.8, *p* = 0.047). Regarding the 5-HT_2_ receptor (Fig 4C and 4D), in the brainstem, decreased values were identified in both sexes (Fig 4D, *F*_(1,19)_ = 4.6, *p* = 0.045).

**Fig 5:**
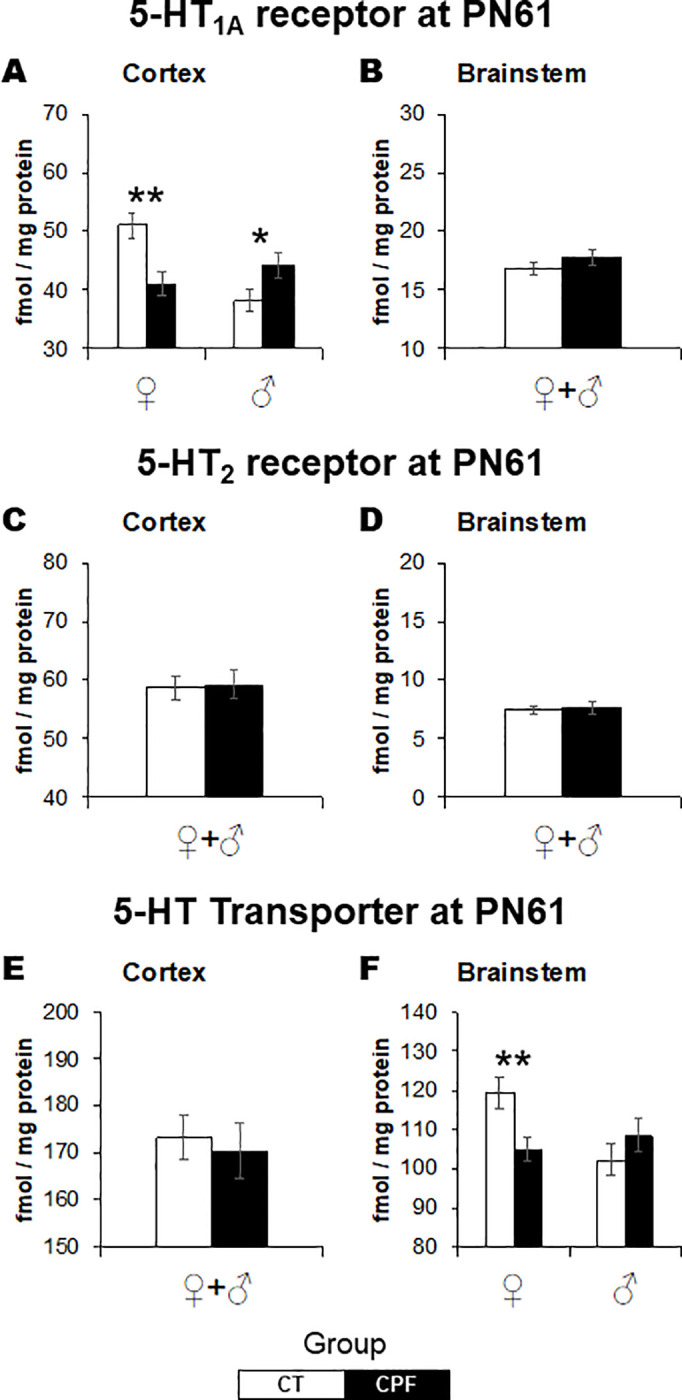
Effects of CPF exposure during the brain growth spurt (3 mg/Kg/day s.c., from PN3 to PN9) on the serotonergic system at adulthood. 5-HT1a in the cerebral cortex (A) and brainstem (B), 5-HT2 in the cerebral cortex (C) and brainstem (D), and 5-HT transporter in the cerebral cortex (E) and brainstem (F). 5-HT1a, serotonin receptor subtype 1a. 5-HT2, serotonin receptor subtype 2; 5-HT, serotonin. For each treatment group, 16 animals were examined, divided into males and females. White bars represent the control group and gray bars represent the CPF-exposed ones. Values are means ± SEM. *p < 0.05, **p < 0.01, versus respective control group.

CPF exposure elicited late-emergent sex-dependent effects in 5-HT_1a_ receptor binding in the cerebral cortex (*Treatment x Sex*: *F*_(1,29)_ = 16.1, *p* < 0.001). As described for ChAT activity, for 5-HT_1a_ there were opposing effects in adult males and females (Fig 5A). While in adult females, 5-HT_1a_ receptor binding was reduced (*Treatment*: *F*_(1,159)_ = 12.2, *p* = 0.004), it was increased in males (*Treatment*: *F*_(1,15)_ = 4.7, *p* = 0.048). No alterations were observed in the brainstem. Regarding the 5-HT_2_ receptor, there were no lasting effects in both studied regions at adulthood (Fig 5C and 5D). As for the 5-HT transporter binding, sex-dependent effects were identified again but restricted to the brainstem (*Treatment x Sex*: *F*_(1,29)_ = 7.2, *p* = 0.012), where CPF increased 5-HT transporter binding in female mice only (*Treatment*: *F*_(1,15)_ = 9.1, *p* = 0.009) (Fig 5F). There were no alterations in the cerebral cortex (Fig 5E).

## Discussion

In the present study, CPF exposure during the brain growth spurt failed to evoke significant alterations in AChE activity in both the cerebral cortex and brainstem at the end of the exposure period. The cumulative dose of the daily injections administered in our model suggests that the peak of inhibition occurs at PN9. However, there is evidence that rodents during gestation are capable of a fast AChE synthesis between each daily dose [34], which suggests that early exposure leads to less severe AChE inhibition than comparable exposures later in life. Considering this possibility, AChE activity levels identified in CPF animals most likely reflect the cumulative effects of the irreversible AChE inhibition generated by multiple exposures that is not compensated by the enzyme synthesis that might occur between successive re-exposures. Our experimental design does not allow us to verify whether enzyme synthesis play an important role in determining AChE activity levels, however, it should be noted that Lassiter and colleagues (1998) demonstrated that AChE activity in the fetal brain only recovers to control levels 24 h after the last dose of a repeated CPF dosing schedule [34]. They also showed that a single CPF dose does not produce statistically significant inhibition of AChE in the fetus’ brain, suggesting that a repeated dosing schedule is effective in inhibiting brain AChE activity. In this sense, we believe that the lack of significant AChE inhibition at PN9 is a good indication that our dose regimen represents a very low-level of CPF exposure in mice. Our data also indicate a programming effect of early CPF exposure since both the cholinergic and serotonergic effects that were observed shortly after exposure were dissimilar to the alterations observed later in life. In the present study, we demonstrated that early CPF exposure increased anxiety-like behavior in females and increased time spent in the center of the EPM in both sexes. The ensuing paragraphs will discuss these behavioral alterations mainly in light of the identified cholinergic and serotoninergic disruptions.

Sex-selective actions of CPF may be associated with its potential actions as an endocrine disruptor. In general, OPs were shown to affect pituitary and sex hormones levels [35], which may distinctively affect females when compared to males. In addition, CPF exposure in the early postnatal period reduces aromatase activity, affecting the biosynthesis of estrogens [36]. A further investigation on the effects of these and other hormonal alterations elicited by CPF may be relevant to explain the increased anxiety-like behavior and biochemical alterations observed in females.

Interestingly, female mice showed transient alterations in the cholinergic and serotonergic systems at the end of CPF exposure. At PN10, both serotonin and choline transporter bindings were increased in the cerebral cortex. These results suggest more intense synaptic activity in response to CPF exposure. Since both serotonergic and cholinergic systems are the important players in the mechanisms of neurogenesis and neuroplasticity [37, 38], these CPF alterations on serotonin and choline transporter binding may profoundly impact cortical development. In addition, CPF exposure in females increased 5-HT_1a_ receptor binding in the brainstem. Taken together, these results reinforce the multiplicity of effects of CPF during development and their capacity to sex-selectively affect the cholinergic and serotonergic systems. Most importantly, these effects could program changes that emerge later during development or at adulthood. In our model, low dose CPF exposure during the brain growth spurt has long-term impact on anxiety-like levels in females. In parallel, all cholinergic and serotonergic alterations that were identified in adult females indicate a hypoactive state.

In this regard, ChAT activity is reduced in the brainstem. Since ChAT is a constitutive marker of cholinergic synapses [23, 39], decreased ChAT activity indicates loss of cholinergic innervation. A reduction in cholinergic innervation in the brainstem may result in a poor control of the mesocorticolimbic pathway activity, since the ventral tegmental area receives cholinergic input mainly from the pedunculopontine and laterodorsal tegmental nuclei [40]. Accordingly, this reduction may contribute to long-term anxiogenic effects in CPF females. On the other hand, CPF males seem to show a compensatory sprouting of cholinergic terminals in brainstem and do not show any alterations on anxiety-like behavior.

The serotonergic alterations identified at adulthood may also have contributed to the anxiogenic profile of CPF females. Regarding the 5-HT transporter, the reduced binding may exacerbate anxiety levels due to an associated increase of 5-HT in the synaptic clefts. In fact, activation of serotonergic neurons in the median raphe nucleus is correlated to enhanced anxiety-like behavior in mice [41]. In addition, 5-HT_1a_ receptor binding was reduced in the cerebral cortex. Alterations in cortical 5-HT_1a_ receptor are associated with mood disorders [42, 43]. Accordingly, mutant mice lacking functional 5-HT_1a_ receptors exhibit elevated levels of anxiety-like behavior [44]. Considering the heterogeneity of 5-HT1_a_ receptor distribution in the cerebral cortex, future studies specifically assessing the modulation of this receptor are essential to better understand the role of the serotonergic system in mood disorders associated with developmental CPF exposure.

Despite the higher susceptibility of females, early exposure to CPF also affected some biomarkers in male mice. 5-HT_2_ receptor binding was reduced in brainstem at PN10 and the time in the center of the EPM was increased in both sexes. It has been suggested that the 5-HT_2_ receptor is a positive modulator of serotonergic tone and that it acts on 5-HT neuron excitability [45]. In this sense, alterations in 5-HT_2_ receptor expression could have profound effects on serotoninergic function and, consequently, evoke long lasting alterations in behavior. Here, CPF adult mice showed an increase in time spent in the center of the EPM. Despite the fact that there is no consensus regarding the meaning of this measure, the time in the center of the maze may reflect decision making, perhaps related to approach/avoid conflict [20, 46]. The proposal is based by the idea that the animal choice between two alternatives (in this case the open and closed arms of the maze) is as a cognitive function based on information collected from the environment. Under this perspective, our data may reflect a reduction in impulsivity levels and/or cognitive function. In adult rats, repeated exposure to CPF leads to sustained attention impairment and increased impulsive behaviors [47, 48]. Developmental CPF exposure was shown to elicit long-lasting impairment in cognition in a preclinical study [49]. In line with this finding, several epidemiological studies have found associations between OPs, including CPF, and cognitive impartments during child development [50, 51]. Accordingly, our data most likely reflect a CPF-induced impairment in cognitive function, expressed as increased time spent in the cognitive task of choosing which arm (open or closed) to go into.

## Conclusions

Our results indicate that CPF exposure during the brain growth spurt affects parameters of brain development which could be related to mood disorders. Since most alterations were identified in females, it is essential to investigate the mechanisms related to this sex-dependent vulnerability. In addition, our data indicate that CPF may act on distinct neurotransmitter systems through distinct mechanisms. Furthermore, dissimilar immediate and late-emergent effects reinforce the idea that distinct mechanisms of action are responsible for both the immediate and the delayed effects. Taken together, the present study clearly evidence the harmful effects of developmental exposure to low doses of CPF and highlights the urgent need for a review of the practices and legislation regarding the use of this pesticide.

## Abbreviations

AChE: acetylcholinesterase
ANOVA: analysis of variance
CPF: chlorpyrifos
ChAT: choline acetyltransferase
Ch transporter: high-affinity presynaptic choline transporter
CT: control
DTNB, 5: 5-dithiobis(2-nitrobenzoic) acid
FPLSD: Fisher’s Protected Least Significant Difference
OP: organophosphate
PN: postnatal day
